# 

**Published:** 1889-02

**Authors:** 


					﻿Vol. IX.
FEBRUARY, 1889.
NO.Z
JUHE
Homeopathic phy^icia]\i,
A Monthly Journal of Homoeopathic Materia Medica
and Clinical Medicine.
“ He is a freeman whom truth makes free, and all are slaves beside."
“Seek the Truth: come whence it may, cost what it will."
EDITED AND PUBLISHED BY
Edmund j. lee, m. e>.,
AND
WALTER M; JAMES, M. E>,
PHILADELPHIA :
No. 11©3 SPRUCE 8TR.B3HJ0?;
T.Q JST X> O2STI
ALFRED HEATH & CO., SOLE AGENTS POE GREAT BRITAIN,
H4 Ebury Street, Eaton Square.
-83“ Yearly Suftecriptiow, $2.SO. invariably in advance. Single numbers, 9S cts.
Entered at the Philadelphia Poet-Office as Second-Class Mail Matter.
				

